# AGEs–RAGE Axis and Mitochondrial-Derived Peptides in Advanced Chronic Kidney Disease: Interconnected Pathways Beyond Inflammation and Nutrition

**DOI:** 10.3390/antiox15080956

**Published:** 2026-07-30

**Authors:** Mohamed E. Suliman, Awahan Rahman, Abdul-Rashid Qureshi, Peter Barany, Peter Stenvinkel, Karolina Kublickiene, Bengt Lindholm

**Affiliations:** Renal Medicine, Department of Clinical Sciences, Intervention and Technology, Karolinska University Hospital Huddinge, Karolinska Institutet, 14186 Stockholm, Sweden; mohamed.saeid.suliman@ki.se (M.E.S.); awahan.reheman@ki.se (A.R.); tony.qureshi@ki.se (A.-R.Q.); peter.barany@ki.se (P.B.); peter.stenvinkel@ki.se (P.S.); karolina.kublickiene@ki.se (K.K.)

**Keywords:** AGE–RAGE axis, mitochondrial-derived peptides, protein-energy wasting, inflammation, chronic kidney disease, kidney transplant

## Abstract

**Background.** Chronic kidney disease [CKD] is characterized by increased glycoxidative stress, inflammation, and mitochondrial dysfunction. Advanced glycation end-products [AGEs] and their receptor [RAGE] mediate glycoxidative stress, whereas mitochondrial-derived peptides [MDPs], including humanin [HN], MOTS-c, and humanin-like 1 [HN-L1], regulate mitochondrial stress responses. We investigated links between AGE–RAGE activation and mitochondrial signaling in CKD. **Methods.** Serum AGEs, soluble RAGE isoforms, and MDPs were measured by ELISA in 160 adults with kidney failure undergoing living-donor kidney transplantation and in 80 controls, **Results.** CKD patients showed higher AGEs and esRAGE, lower AGEs/sRAGE ratios, and reduced MDPs. AGEs correlated positively with sRAGE, cRAGE, and HN, while MOTS-c was inversely associated with AGEs and the AGEs/sRAGE ratio. In multivariable analyses, HN remained independently associated with AGEs, sRAGE, and the AGEs/sRAGE ratio, whereas MOTS-c showed an inverse association with the AGEs/sRAGE ratio. CRP was associated with the AGEs/sRAGE ratio while PEW showed no associations with AGEs–RAGE components or MDP. **Conclusions.** CKD is characterized by increased glycoxidative stress and reduced MDPs, reflecting altered mitochondrial stress signaling. AGEs–RAGE and MDPs are biologically linked, suggesting partially overlapping but distinct pathophysiological pathways.

## 1. Introduction

Chronic kidney disease (CKD) is a progressive condition characterized by metabolic disturbances, chronic inflammation, and oxidative stress, all of which contribute to increased cardiovascular morbidity and mortality. Among the molecular mediators implicated in CKD, advanced glycation end products (AGEs) and their receptor (RAGE) play a pivotal role. AGEs are formed through non-enzymatic glycation of proteins and lipids and accumulate in uremic conditions due to both increased formation and reduced renal clearance [1,2,3]. Activation of the AGE–RAGE axis promotes reactive oxygen species (ROS) generation, inflammation, and endothelial dysfunction [4], all which are implicated in CKD and cardiovascular complications.

In addition to membrane-bound full-length RAGE, two major soluble forms of RAGE (sRAGE) exist: cleavage RAGE (cRAGE) and endogenous secretory RAGE (esRAGE) [5,6]. These soluble variants lack transmembrane and cytoplasmic domains and are found in circulating blood and body fluids [5,6]. Acting as decoy receptors, sRAGE binds circulating RAGE ligands and prevents their interaction with membrane-bound RAGE, thereby attenuating RAGE-mediated signaling, cellular activation, and inflammation [7]. cRAGE is produced through proteolytic cleavage of membrane-bound RAGE, whereas esRAGE, generated by alternative splicing, accounts for less than 25% of circulating sRAGE [6]. Circulating sRAGE has been proposed as a biomarker of disease risk and adverse outcomes and may have therapeutic potential in RAGE-related disorders [5,6,8].

In parallel, mitochondrial dysfunction has emerged as a key contributor to CKD progression [9]. Mitochondria produce mitochondrial-derived peptides (MDPs), which are translated peptides encoded by short open reading frames (sORFs) within mitochondrial DNA (mtDNA) genes and mediate the transcriptional stress response by translocating into the nucleus and interacting with DNA [10]. MDPs, such as humanin (HN), mitochondrial open reading frame of the 12S rRNA-c (MOTS-c), and humanin-like-1 peptides (HN-L1), are increasingly recognized for their cytoprotective, anti-apoptotic, and metabolic regulatory properties [11,12,13]. As the biological functions of HN-L1 are less well characterized than those of HN and MOTS-c, emerging evidence suggests that humanin-like peptides participate in cytoprotective signaling and mitochondrial stress responses [10]. Therefore, HN-L1 may offer additional insight into mitochondrial stress responses and biomarker potential in CKD. Emerging evidence suggests that mitochondrial health and glycation burden are closely interconnected. AGEs and activation of their receptor promote the generation of ROS, leading to mtDNA damage and impaired oxidative phosphorylation [14]. In turn, mitochondrial dysfunction amplifies oxidative stress and glycoxidative injury [15,16], establishing a self-reinforcing cycle.

Although mitochondrial dysfunction and AGE accumulation are well documented in patients with CKD [2,17,18], no clinical studies have specifically examined the relationship between MDPs and AGEs in this population. Experimental studies showed that AGEs can directly induce mitochondrial dysfunction [19,20,21], suggesting a bidirectional link between glycation stress and mitochondrial impairment. Notably, both AGE accumulation and mitochondrial dysfunction are recognized hallmarks of ageing [3,22], suggesting that alterations in MDP signaling may contribute to the accelerated ageing phenotype observed in CKD [23]. However, despite this mechanistic plausibility, clinical evidence integrating AGEs, RAGE isoforms, and MDPs in CKD patients remains lacking. Moreover, dysregulation of these interconnected pathways may contribute to the heightened cardiovascular burden associated with CKD, including endothelial dysfunction, vascular calcification, and accelerated vascular ageing.

Therefore, this study aimed to investigate the interrelationships between circulating AGEs–RAGE axis components and mitochondrial-derived peptides (MDPs), and to compare their levels between non-CKD subjects and adult CKD patients undergoing living-donor kidney transplantation (LD-KTx). In addition, we explored associations among these biomarkers and clinical parameters, with particular attention to protein-energy wasting (PEW) and inflammation, both of which are highly prevalent in CKD and may have biologically plausible links to these pathways.

## 2. Material and Methods

### 2.1. Materials

This study is a post hoc analysis of baseline data derived from an ongoing prospective cohort of adult patients with CKD stage 5 (CKD-5) undergoing LD-KTx at the Department of Transplantation Surgery, Karolinska University Hospital, between 2009 and 2022 [24]. Exclusion criteria included age < 18 years, acute kidney injury, clinical evidence of overt infection, and unwillingness to participate. Accordingly, this work should be considered an observational cross-sectional study. For the purposes of the present analysis, 160 of the 205 patients in the cohort were included, based exclusively on the availability of baseline measurements of serum concentrations of AGEs, RAGE isoforms, and MDPs. Baseline characteristics are summarized in Table 1.

The CKD patients had a median age of 47 years, and 68% were men. The median estimated glomerular filtration rate (eGFR) was 6.4 mL/min/1.73 m^2^ (interquartile range (IQR): 5.2–8.4). The medications among the CKD-5 patients before the kidney transplantation (KTx) were antihypertensive medications (n = 136, 85%), erythropoietin (n = 130, 81%), statins (n = 56, 35%), phosphate binders (n = 149, 93%) and sodium bicarbonate (n = 66, 41%). Moreover, according to the LD-KTx protocol, recipients commenced treatment with prednisolone and mycophenolate mofetil two days prior to transplantation.

A community-dwelling control [CDC] group [n = 80; 71% men; median age 63 years; median eGFR 81 mL/min/1.73 m^2^] were included for comparative analyses of circulating AGEs, sRAGE isoforms, and MDPs measurements. This group consisted of randomly selected individuals from a population-based cohort identified by Statistics Sweden [www.scb.se; accessed on 10 October 2006] and recruited in the Stockholm region between February 2003 and September 2006.

Written informed consent was obtained from all participants. The studies protocol was approved by the Swedish Ethical Review Authority (Dnr 2008/1748-31/2; date of approval 27 November 2008 and Dnr 2010/579-31; date of approval 4 June 2010).

### 2.2. Methods

Plasma and serum blood samples were collected from the LD-KTx patients and CDC subjects in the morning after an overnight fast. In CKD patients, blood samples were drawn on the day before LD-KTx. The serum and plasma blood samples from patients and controls were kept frozen at −70 °C pending analyses, if not analyzed immediately.

ELISA kits from AssayGenie [Dublin, Ireland] were used to assay serum AGEs [CAT# HUFI00449], sRAGE [CAT# HUFI02967], MOTS-c [CAT# AEFI00422], and HN [CAT# HUFI03862]. ELISA kits from CliniSciences [Copenhagen, Denmark] were used to measure serum HN-L1 [CAT# MBS2890008] and esRAGE [CAT# MBS167330]. cRAGE was calculated by subtracting serum concentrations of esRAGE from sRAGE. The intra-assay coefficient of variation (CV), inter-assay CV, and lower limit of detection were, respectively, ≤8%, ≤10%, and 0.188 ng/mL for AGEs; ≤8%, ≤10%, and 18.75 pg/mL for sRAGE; ≤8%, ≤10%, and 2.48 ng/L for esRAGE; ≤8%, ≤10%, and 0.25 pg/mL for MOTS-c; ≤8%, ≤10%, and 18.75 pg/mL for Humanin (HN); and ≤5.4%, ≤7.9%, and 0.35 ng/mL for Humanin-L1 (HN-L1). All biomarker measurements were analyzed by ELISA contemporaneously by a single investigator (AR) and with the same analytical batches to minimize inter-assay variability. Of note, the ELISA kit for AGE measures the total serum concentration of AGEs rather than any specific chemically defined AGE species.

Plasma high-sensitivity C-reactive protein [hsCRP] was measured by nephelometry at the Department of Laboratory Medicine, Karolinska University Hospital, Huddinge, Sweden. Plasma interleukin-6 [IL-6] and tumor necrosis factor [TNF] concentrations were analyzed, using commercial kits on an Immulite Automatic Analyzer [Siemens Medical Solutions, Los Angeles, CA, USA], according to the manufacturer’s instructions. Other biochemical analyses were performed using validated routine methods at the certified Clinical Laboratory of Karolinska University Hospital [Stockholm, Sweden] or at the Research Laboratory of the Department of Renal Medicine, Karolinska University Hospital Huddinge.

Glomerular filtration rate (GFR) was assessed in the CKD patients as an estimated GFR (eGFR) from serum creatinine levels using the 2021 CKD-EPI creatinine equation without race coefficient [25]. The PEW was evaluated using the four-point of subjective global assessment (SGA) scale for nutritional status and PEW was defined as SGA score > 1 [26].

### 2.3. Statistical Methods

As most variables were not normally distributed, data are presented as medians with interquartile ranges (IQR). Non-parametric tests were used to assess differences in central tendency. The Mann–Whitney U test was applied for comparisons between two groups. Correlation analyses were performed using Spearman’s rank correlation method. Multiple linear regression analyses adjusted for age, sex, CRP, PEW, BMI, and eGFR were performed to assess the associations of AGEs, sRAGE, and MDP concentrations, as well as the association between esRAGE and PEW. All continuous variables included in the regression models were log-transformed and standardized prior to analysis. Results are presented as a volcano plot showing effect sizes (log_2_ fold changes) and statistical significance (−log_10_ *p*-values) derived from the multivariable regression analyses. Given the exploratory nature of the analyses, adjusting for multiple comparison between variables was not used and findings should be considered hypothesis-generating. All statistical tests were two-tailed, and a *p*-value < 0.05 was considered statistically significant. All statistical analyses were conducted using Stata 19.0 (Stata Corporation, College Station, TX, USA) and SAS version 9.4 service 8 (SAS Campus Drive, Cary, NC, USA).

## 3. Results

### 3.1. Characteristics of the CKD Patients

Of the 205 CKD stage 5 patients undergoing living-donor renal transplantation (LD-RTx) in the cohort, 160 were included in the present study based exclusively on the availability of baseline serum measurements of AGEs, RAGE isoforms, and MDPs. The excluded patients (n = 45) were generally comparable to those included with respect to clinical characteristics, with the exception of a lower prevalence of CVD and higher hemoglobin in the excluded group (Table S1). Among the 160 included, 109 (68%) were male. Of these patients, 54 (34%) underwent preemptive RTx without prior dialysis, while 57 (36%) received hemodialysis (HD) and 49 (30%) underwent peritoneal dialysis (PD) before RTx.

The median (IQR) age was 47 (32–58) years. Median s-creatinine did not differ significantly in dialyzed (723 μmol/L) versus non-dialyzed (730 μmol/L) patients. Among those who underwent dialysis, the median vintage duration was 12.1 (5.7–21.7) months, with no significant difference between HD and PD (14.4 [5.0–25.1] vs. 11.5 [7.4–14.8] months; *p* = 0.22), respectively.

The CKD patient cohort had a median eGFR of 6.1 (range: 3.4–18.4), mL/min/1.73 m^2^, with no significant difference in the median between dialyzed and non-dialyzed (6.31 vs. 6.40, mL/min/1.73 m^2^, respectively). Seventeen patients (11%) had DM, 26 (16%) had CVD, and 40 (26%) were malnourished. These data, along with other demographic and clinical characteristics, are summarized in Table 1.

### 3.2. Serum Concentrations of AGEs, sRAGE Isoforms and MDPs in the CKD Patients and Control Subjects

**Figure 1:**
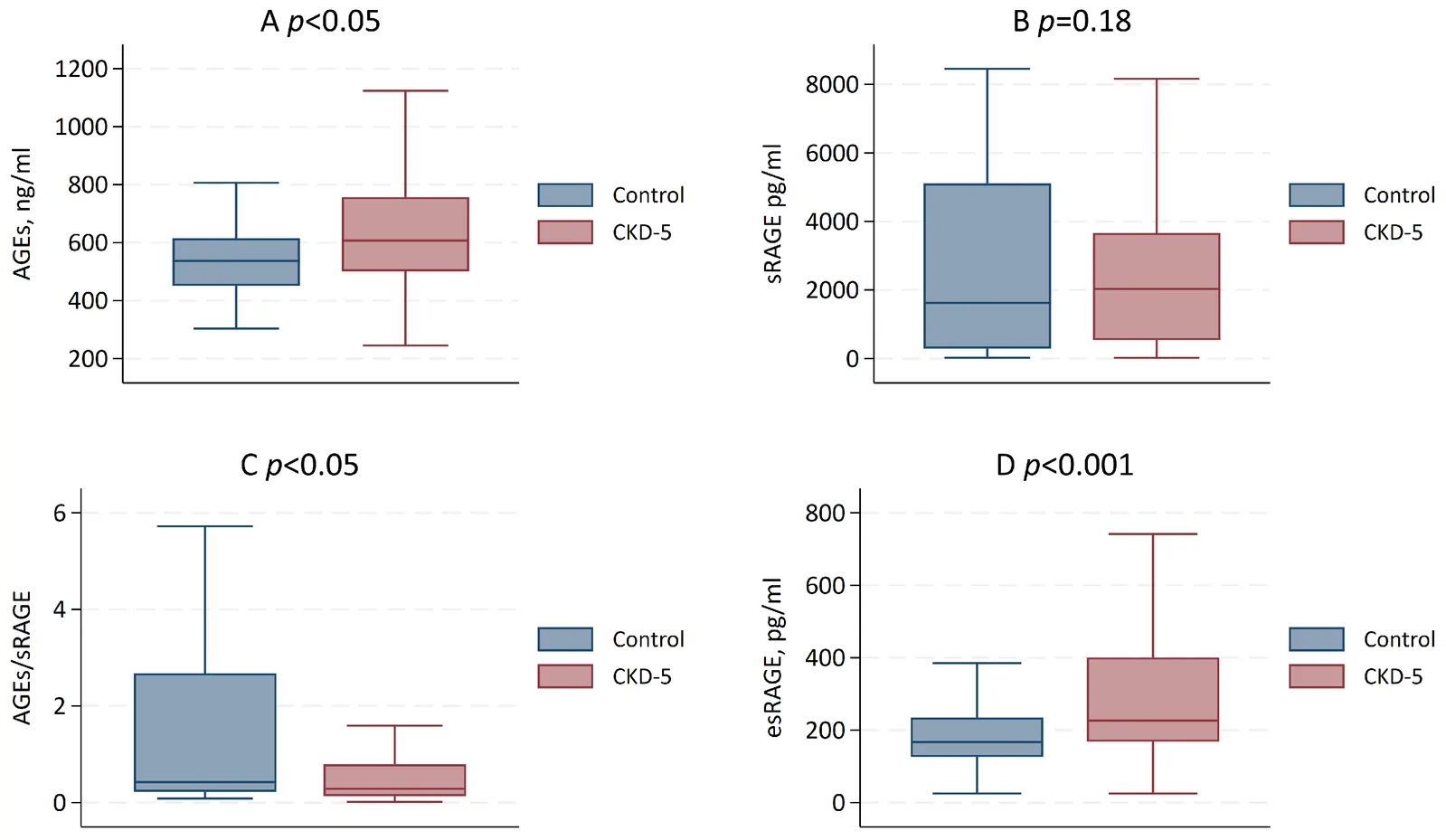
Box plots representing variations in serum concentrations of (**A**) advanced glycation end products (AGEs), (**B**) soluble receptor for advanced glycation end products (sRAGE), (**C**) AGEs/sRAGE ratio, and (**D**) endogenous secretory receptor for AGEs (esRAGE) in control subjects and CKD-5 patients.

**Figure 2:**
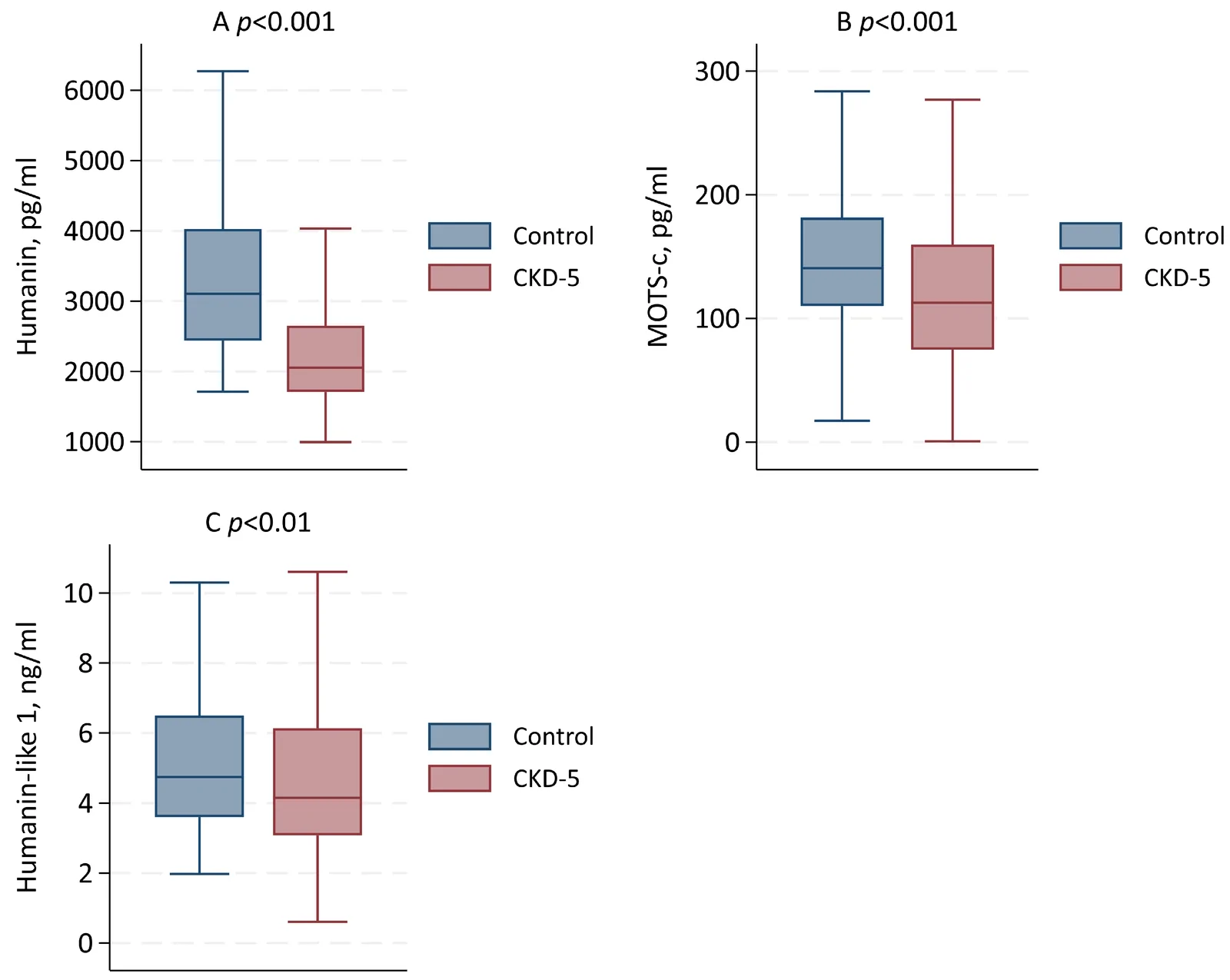
Box plots comparing serum concentrations of mitochondrial-derived peptides including (**A**) humanin, (**B**) mitochondrial open reading frame of the 12S rRNA-c peptide (MOTS-c) and (**C**) humanin-like 1 in control subjects and CKD-5 patients.

A comparative analysis revealed distinct biomarker profiles between the CKD patients and the CDC (Figure 1 and Figure 2 and Table S2a). The CKD patients exhibited significantly higher levels of AGEs and esRAGE and lower AGEs–sRAGE ratio compared with CDC (Figure 1 and Table S2a), indicating increased glycoxidative stress. sRAGE and cRAGE showed a non-significant trend towards being higher in CKD (Figure 1 and Table S2a). Conversely, all measured MDPs (HN, MOTS-c, and HN-L1) were significantly reduced in CKD patients (Figure 2 and Table S2a), suggesting impaired mitochondrial signaling. Of note, because the control subjects were older than the CKD patients, we conducted a separate analysis using only control subjects below the median age. The findings were essentially unchanged from those obtained with the full control cohort, except that the lower MOTS-c concentration observed in CKD patients was no longer statistically significant (Table S2b). Moreover, the study did not show significant differences in the levels of AGEs, sRAGE isoforms and MPDs between the dialyzed and non-dialyzed CKD patients (Table S3).

### 3.3. Age- and Sex-Related Associations

Tables S4a and S4b present the associations of age and sex, respectively, with AGEs, sRAGE isoforms, and MDPs in patients and control subjects.

Age (Table S4a) was not significantly correlated with any of the measured variables in the patient group (all *p* > 0.05). In contrast, several significant associations were identified in the control group (Table S4a). Specifically, HN showed a negative correlation with age (rho (ρ) = −0.24, *p* = 0.04), whereas MOTS-c demonstrated a positive correlation (ρ = 0.25, *p* = 0.03). In addition, the AGEs/RAGE ratio was positively correlated with age in controls (ρ = 0.39, *p* = 0.008). No significant correlations with age were observed for AGEs, sRAGE, esRAGE, cRAGE, or HN-L1 in either group.

Sex (Table S4b) was not significantly correlated with any of the measured variables in the patient group (all *p* > 0.05), whereas majority of variables differed significantly between men and women in the control group.

### 3.4. Correlations Between AGEs, RAGE Isoforms and MDPs

As shown in Table 2, in LD-RTx patients, AGEs were significantly and positively correlated with sRAGE (ρ = 0.28, *p* = 0.006), cRAGE (ρ = 0.25, *p* = 0.02), and the cRAGE/esRAGE ratio (ρ = 0.23, *p* = 0.03). A non-significant trend toward a negative association was observed between AGEs and esRAGE (ρ = −0.15, *p* = 0.09). Humanin was negatively correlated with MOTS-c (ρ = −0.22, *p* = 0.02) and HN-L1 (ρ = −0.33, *p* = 0.0003), whereas MOTS-c and HN-L1 were positively correlated (ρ = 0.28, *p* = 0.0003).

Moreover, HN showed significant and positive associations with AGEs (ρ = 0.41, *p* < 0.0001), sRAGE (ρ = 0.30, *p* = 0.005), cRAGE (ρ = 0.35, *p* = 0.002), and the cRAGE/esRAGE ratio (ρ = 0.39, *p =* 0.0004), along with a negative association with the AGEs/sRAGE (ρ = −0.21, *p* = 0.04) and a non-significant trend toward a negative association with esRAGE (ρ = −0.17, *p* = 0.08). In contrast, MOTS-c was negatively correlated with AGEs (ρ = −0.22, *p* = 0.009) and the AGEs/sRAGE ratio (ρ = −0.20, *p* = 0.04) and positively correlated with the cRAGE/esRAGE ratio (=0.21, *p* = 0.03). A non-significant positive trend was observed between MOTS-c and sRAGE (ρ = 0.18, *p* = 0.07). No significant associations were observed between HN-L1 and AGEs or sRAGE isoforms.

### 3.5. Independent Associations Between AGEs–RAGE Axis and MDPs

**Figure 3:**
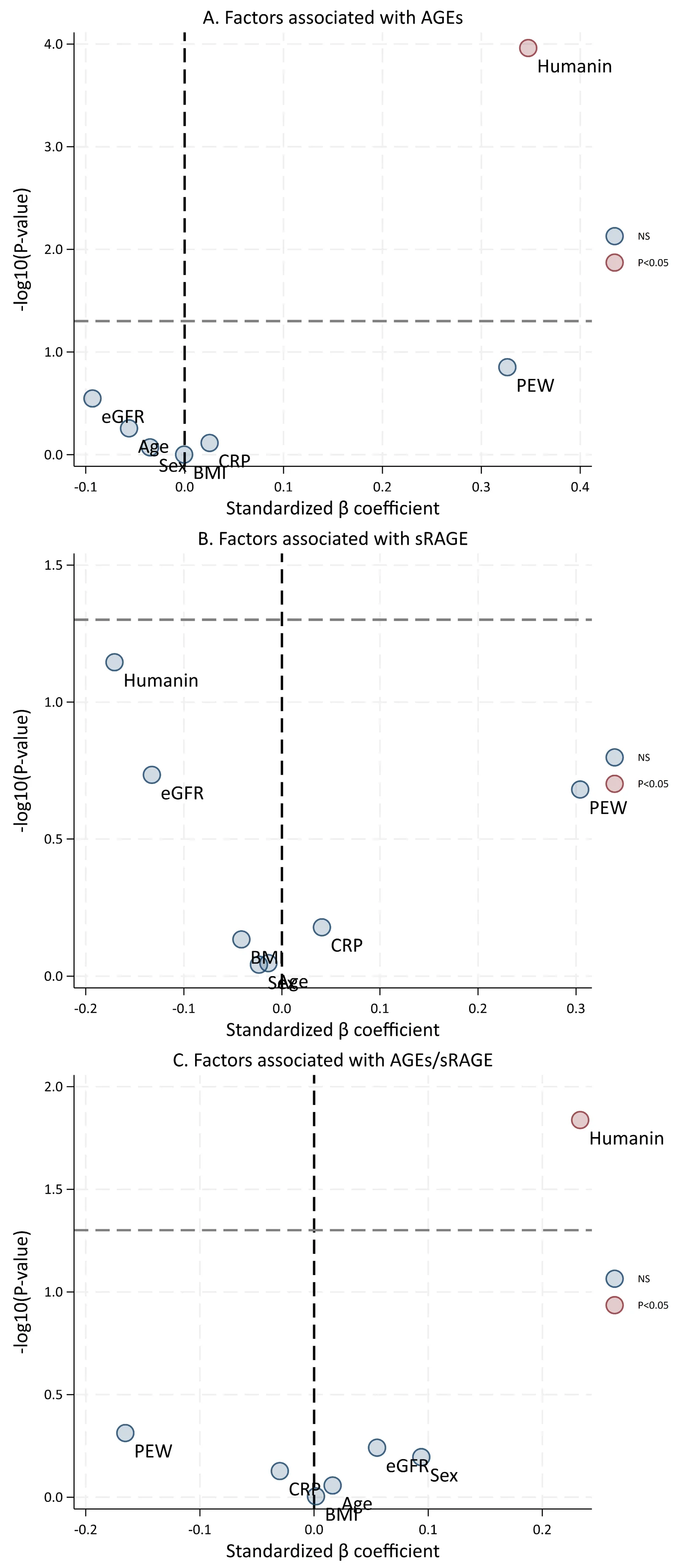
Volcano plots showing factors associated with (**A**) advanced glycation end products (AGEs), (**B**) soluble receptor for advanced glycation end products (sRAGE), and (**C**) the AGEs/sRAGE ratio in multivariable linear regression analyses. The models included age, sex, C-reactive protein (CRP), protein-energy wasting (PEW) assessed by the subjective global assessment (SGA), body mass index (BMI), humanin, and glomerular filtration rate (GFR) as independent variables. Red and blue dots represent statistically significant and non-significant standardized regression coefficients (β), respectively. Humanin was the only variable independently associated with all three outcomes (AGEs, sRAGE, and the AGEs/sRAGE ratio) (additional details are provided in Supplementary Table S5a–c).

To assess interrelationships among AGEs, sRAGE and MDPs, we conducted a series of multivariate linear regression analyses. First, we examined associations with AGEs as the dependent variable and age, sex, CRP, PEW (assessed by SGA), BMI, HN, and GFR as independent variables (Figure 3 and Table S5a). Among the tested covariates, only HN was the independent predictor and significantly associated with AGEs (β = 0.35, SE = 0.087, *p* < 0.0001, 95% CI: 0.176–0.523), indicating a positive relationship; no other associations with covariates were significant.

We then fitted the same model with sRAGE as the dependent variable with same set of covariates (Figure 3 and Table S5b). Again, HN was the only significant predictor (β = 0.30, SE = 0.096, *p* = 0.003, 95% CI: 0.106–0.492).

Finally, using the AGEs/sRAGE ratio as the dependent variable and the same set of covariates (Figure 3 and Table S5c), HN remained the only significant predictor, showing a negative association with the ratio (β = −0.25, SE = 0.10, *p* = 0.016, 95% CI: −0.454–−0.048).

**Figure 4:**
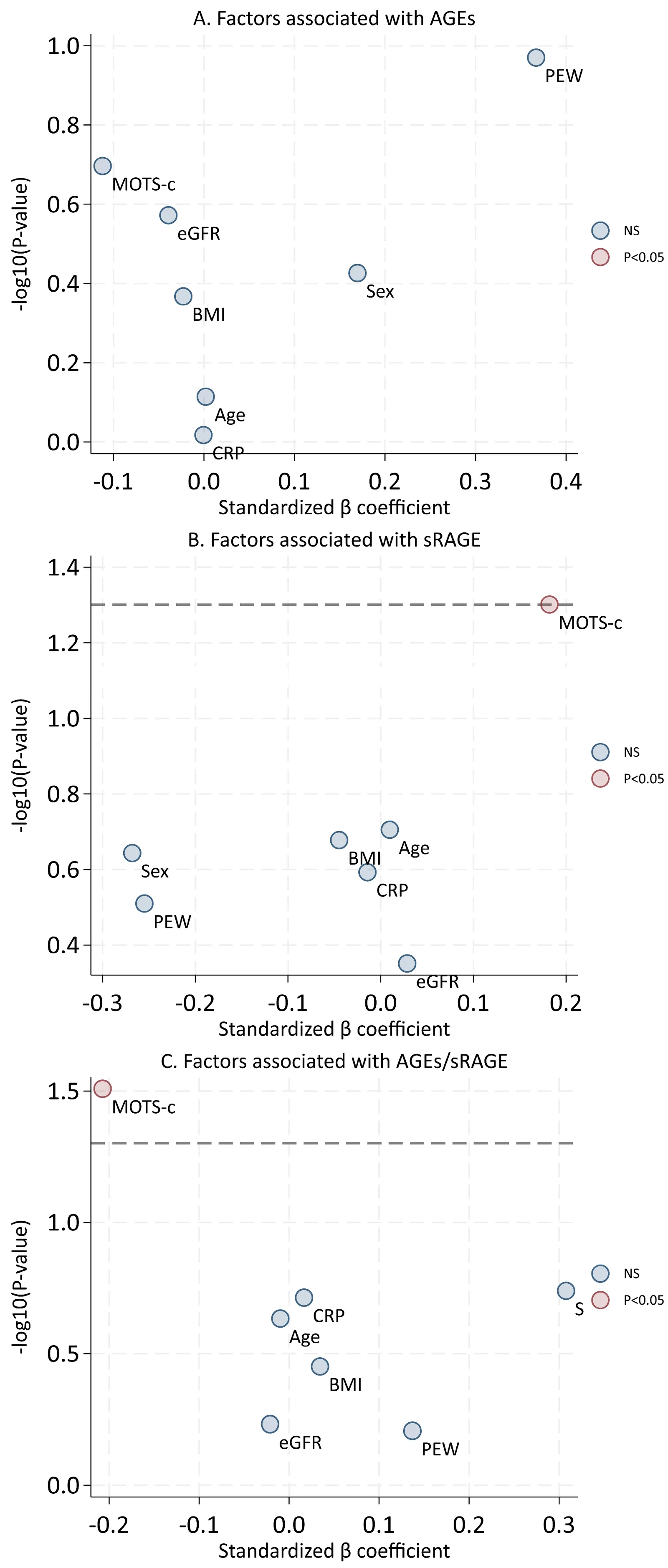
Volcano plots showing factors associated with (**A**) advanced glycation end products (AGEs), (**B**) soluble receptor for advanced glycation end products (sRAGE), and (**C**) the AGEs/sRAGE ratio in multivariable linear regression analyses in models including MOTS-c and not humanin. The models included age, sex, C-reactive protein (CRP), protein-energy wasting (PEW) assessed by the Subjective Global Assessment (SGA), body mass index (BMI), mitochondrial open reading frame of the 12S rRNA-c peptide (MOTS-c), and glomerular filtration rate (GFR) as independent variables. Red and blue dots represent statistically significant and non-significant standardized regression coefficients (β), respectively. MOTS-c was independently associated with sRAGE and the AGEs/sRAGE ratio, but not with AGEs. In addition, the AGEs/sRAGE ratio was independently associated with CRP (additional details are provided in Supplementary Table S6a,c).

Using the same regression models, with HN replaced by MOTS-c, we assessed whether MOTS-c was associated with AGEs, sRAGE, and/or the AGEs/sRAGE ratio (Figure 4 and Table S6). The AGEs/sRAGE ratio was independently and negatively associated with MOTS-c (β = −0.207, SE = 0.094, *p* = 0.031; 95% CI: −0.395 to −0.019) (Figure 4 and Table S6c), and MOTS-c showed a borderline significant positive association with sRAGE (β = 0.182, SE = 0.092, *p* = 0.049; 95% CI: 0.0001 to 0.364) (Figure 4 and Table S6b). In contrast, MOTS-c was not significantly associated with AGEs (β = −0.112, SE = 0.087, *p* = 0.20; 95% CI: −0.284 to 0.060) (Figure 4 and Table S6a). Additionally, no other covariates were significantly associated with AGEs, sRAGE, or the AGEs/sRAGE ratio.

### 3.6. Associations with Protein-Energy Wasting

Based on SGA, 40 CKD patients [25%] were classified as having PEW. In PEW patients, esRAGE levels were significantly higher compared with nourished individuals [294 [201–562] vs. 208 [162–308], *p* = 0.015], respectively]. In contrast, no significant differences were observed between the two groups for AGEs [*p* = 0.18], sRAGE [*p* = 0.30], cRAGE [*p* = 0.18], MOTS-c [*p* = 0.78], HN [*p* = 0.64], or HN-L1 [*p* = 0.69].

However, the association between esRAGE and PEW was not maintained in the multivariable regression analysis. In a model adjusted for age, sex, SGA, hsCRP, BMI, and GFR, PEW was not identified as an independent predictor of esRAGE levels [β = 0.32, *p* = 0.11] [Table 3].

### 3.7. Associations with Inflammation

To explore the associations between inflammatory markers [CRP, IL-6, and TNF] with AGEs, sRAGE isoforms, or MDPs in CKD patients, univariate analyses revealed that only CRP was significantly positively correlated with the AGEs/sRAGE ratio [ρ = 0.21, *p* = 0.04] and MOTS-c [ρ = 0.18, *p* = 0.02] in univariate analysis. Furthermore, in multivariable analyses, CRP remained independently associated with the AGEs/sRAGE ratio [β = 0.44, *p* = 0.047] in the model that included MOTS-c as an independent variable [Figure 4 and Table S6c], but not in the model including HN [Figure 3 and Table S5c]. No significant correlations were observed between inflammatory markers and serum levels of AGEs, sRAGE, esRAGE, cRAGE, HN, or HN-L1.

## 4. Discussion

We demonstrate that CKD-5 undergoing LD-Ktx are characterized by elevated circulating concentrations of AGEs and sRAGE isoforms together with significantly reduced levels of MDPs. To our knowledge, we are the first to evaluate the relationships between AGEs, RAGE isoforms, and MDPs in patients with advanced CKD. The findings are consistent with the concept that glycoxidative stress and mitochondrial dysfunction are biologically interconnected processes in CKD and strengthen the suggestion that alteration of circulating MDPs may reflect abnormal mitochondrial homeostasis associated with AGE–RAGE activation [27].

The CKD patients exhibited higher circulating concentrations of AGEs and esRAGE and a nonsignificant trend of higher sRAGE compared with control subjects, while the AGEs/sRAGE ratio was lower. In contrast, all measured MDPs, including HN, MOTS-c, and HN-L1, were obviously reduced in CKD patients, a finding that remained evident even when compared with younger control subjects. These observations are consistent with previous evidence demonstrating enhanced accumulation of AGEs in CKD due to both increased endogenous production and impaired renal clearance [1,2,3]. Notably, dietary intake represents another potential determinant of circulating AGE concentrations, as exogenous AGEs from heat-processed foods can contribute to the systemic AGE pool. Because dietary AGE intake was not assessed in this study, its influence on circulating AGE levels cannot be excluded. Indeed, AGE accumulation progresses with declining renal function, independent of diabetes status, and with further amplification in dialysis patients through exposure to glucose degradation products and reactive dicarbonyl compounds derived from dialysis fluids [28,29,30]. Notably, in the present study, no significant differences were observed in serum concentrations of AGEs, sRAGE isoforms or MDPs between patients receiving dialysis and those who were not on dialysis, suggesting that advanced kidney dysfunction itself, rather than dialysis exposure per se, is the major determinant of the observed biomarker alterations. Accordingly, the elevated AGE levels observed in the present CKD cohort likely reflect the combined effects of reduced renal elimination, chronic oxidative stress, and persistent metabolic disturbances characteristic of advanced CKD. In addition, AGEs may also contribute to CKD progression by impairing endogenous repair mechanisms. Experimental studies have shown that AGEs negatively affect the function, survival, and differentiation capacity of mesenchymal stromal/stem cells, a key component of intrinsic tissue repair processes [31]. Such AGE-mediated impairment of regenerative pathways may further exacerbate tissue injury and limit recovery in CKD, highlighting another potential pathogenic consequence of excessive AGE accumulation [31].

The sRAGE isoforms function as decoy receptors capable of binding circulating AGEs and thereby preventing their interaction with membrane-bound RAGE [28,29,30]. We observed increased esRAGE levels and a trend toward higher sRAGE concentrations in CKD patients. This finding may reflect a combination of altered regulation of the RAGE system and reduced renal clearance, both of which are known to influence circulating sRAGE levels in CKD. Furthermore, the significantly lower AGEs/sRAGE ratio in CKD patients may indicate a relative shift in the balance between ligand availability and decoy receptor levels in the setting of increased glycation burden. However, this ratio is likely to be influenced by both increased AGE formation and impaired renal elimination of sRAGE isoforms. Accordingly, interpretation of circulating sRAGE levels and the AGEs/sRAGE ratio should be undertaken with caution, as they may reflect complex and partially opposing processes, including metabolic stress, receptor shedding, and impaired renal clearance, rather than a single dominant biological mechanism.

In parallel with the alterations in AGE-related biomarkers, CKD patients demonstrated markedly lower levels of all measured MDPs. These findings are consistent with altered circulating MDP profiles that may reflect mitochondrial stress. and reduced cytoprotective capacity in CKD. The present results are partly consistent with a previous study evaluating MDPs in skeletal muscle of CKD patients, in which Liu et al. [32] reported reduced expression of HN and MOTS-c. Mitochondrial dysfunction is increasingly recognized as a key mechanism contributing to CKD progression and associated metabolic complications [9]. MDPs such as HN and MOTS-c exert anti-apoptotic, anti-inflammatory, and metabolic regulatory functions and are thought to participate in adaptive stress responses to mitochondrial injury [11,12,13]. Thus, reduced circulating levels of these peptides may suggest diminished mitochondrial resilience in CKD. However, circulating MDP levels are not solely determined by mitochondrial production and may also be influenced by altered peptide metabolism, degradation, and renal clearance in advanced CKD. Therefore, the observed reductions in circulating MDPs should be interpreted cautiously, as they may reflect a combination of impaired mitochondrial signaling and altered peptide handling associated with kidney dysfunction.

We also observed an interplay between AGEs and sRAGE isoforms with MDPs. AGEs demonstrated significant positive correlations with sRAGE, cRAGE, and the cRAGE/esRAGE ratio, consistent with activation of the AGE–RAGE axis in response to increased glycation burden. This observation concurs with other findings suggesting that elevated circulating RAGE isoforms may potentially be consistent with compensatory responses against AGE-mediated tissue injury [33,34,35]. Interestingly, HN was positively associated with AGEs, sRAGE, and cRAGE, whereas MOTS-c showed inverse associations with AGEs and the AGEs/sRAGE ratio.

The positive association between AGEs and HN may reflect activation of an endogenous cytoprotective mitochondrial stress response. HN has been shown to exert anti-apoptotic and anti-inflammatory actions under conditions of oxidative stress and cellular injury [33,34,35]. Increased glycoxidative stress may therefore stimulate compensatory HN signaling aimed at preserving mitochondrial integrity and cellular survival [12]. In contrast, the inverse relationship between AGEs and MOTS-c may suggest differential regulation of mitochondrial peptides under metabolic stress conditions. MOTS-c primarily regulates metabolic homeostasis, insulin sensitivity, and AMPK-related pathways [27,36,37], while chronic oxidative stress may suppress its production or signaling. These divergent relationships indicate that MDPs are not uniformly regulated in CKD and may participate in distinct adaptive pathways.

The observed interrelationships among MDPs themselves further support the existence of a coordinated mitochondrial signaling network. Humanin was negatively correlated with both MOTS-c and HN-L1, whereas MOTS-c and HN-L1 were positively associated. The inverse associations may reflect differential regulation of individual MDPs in response to mitochondrial stress, suggesting that HN, MOTS-c, and HN-L1 may be activated through distinct adaptive pathways rather than being coordinately regulated. Experimental studies have shown that mitochondrial dysfunction and oxidative stress can activate multiple stress-response pathways simultaneously, with variable downstream metabolic consequences [27]. Thus, the balance between individual MDPs may reflect differential adaptation to mitochondrial injury in CKD.

The multivariable regression analyses further strengthened these observations. Humanin remained independently associated with AGEs, sRAGE, and the AGEs/sRAGE ratio after adjustment for age, sex, inflammation, BMI, nutritional status, and residual kidney function. Similarly, MOTS-c was independently associated with sRAGE and inversely associated with the AGEs/sRAGE ratio. These findings suggest that the observed relationships between MDPs and the AGE–RAGE axis are not simply secondary to renal dysfunction or inflammation but most likely reflect biologically meaningful interactions between mitochondrial stress signaling and AGEs–RAGE axis pathways. Experimental evidence supports such a mechanistic link, as AGEs and RAGE activation promote mitochondrial ROS generation, mitochondrial DNA damage, and impaired oxidative phosphorylation [14] and AGEs can directly induce mitochondrial dysfunction [19,20,21]. In turn, mitochondrial dysfunction amplifies oxidative stress and glycoxidative injury, creating a self-perpetuating cycle [15,16]. The present findings extend this concept to clinically relevant scenario by demonstrating independent associations between AGE-related biomarkers and MDPs in patients with advanced CKD. Although causality cannot be established due to the cross-sectional design, the data suggests that mitochondrial signaling pathways may participate in the systemic response to chronic glycoxidative stress Although causality cannot be established due to the cross-sectional design of this study, the observed associations suggest that mitochondrial signaling pathways may be involved in the systemic response to chronic glycoxidative stress. However, these findings demonstrate associations rather than mechanistic relationships and should not be interpreted as evidence of a direct biological link between the AGE–RAGE pathway and mitochondrial-derived peptides. Alternative explanations should also be considered, including compensatory responses to cellular stress or the parallel activation of distinct stress-response pathways in CKD.

We observed limited associations between AGEs, sRAGE isoforms, MDPs, and classical inflammatory markers such as CRP, IL-6, and TNF. Specifically, CRP was positively associated with the AGEs/sRAGE ratio in both univariate and multivariable analyses. The selective association between the AGEs/sRAGE ratio and CRP, but not IL-6 or TNF, may reflect the greater stability of CRP as a marker of chronic low-grade inflammation, whereas circulating cytokines exhibit greater biological variability influenced by transient inflammatory stimuli. Thus, the AGEs/sRAGE ratio may better capture the cumulative inflammatory burden associated with chronic AGE–RAGE activation in CKD. On the other hand, no significant associations were observed between inflammatory markers and serum levels of AGEs, sRAGE, esRAGE, cRAGE, HN, or HN-L1. In addition, CRP showed a weak positive correlation with MOTS-c in univariate analysis, although this association was not retained in multivariable analysis.

The AGE–RAGE axis is well recognized as a driver of inflammatory signaling [4]. Therefore, the independent association between CRP and the AGEs/sRAGE ratio supports a link between AGE–RAGE pathway activation and systemic inflammation in CKD. Notably, this relationship was observed for the AGEs/sRAGE ratio rather than for AGEs or sRAGE isoforms individually, suggesting that the balance between circulating AGEs and their soluble decoy receptor may better reflect the inflammatory burden associated with AGE–RAGE signaling. At the same time, the generally weak and selective nature of these associations suggests that glycoxidative stress and mitochondrial dysfunction in CKD are not fully captured by conventional circulating inflammatory biomarkers. One explanation is that AGEs and MDPs reflect chronic oxidative and metabolic stress, whereas CRP and cytokines represent more dynamic systemic inflammatory activity influenced by multiple clinical and environmental factors. In addition, inflammation in CKD is often compartmentalized, with local tissue-level inflammatory processes not necessarily mirrored by circulating cytokine concentrations [38,39,40,41]. Another possible explanation is the presence of a ceiling effect in advanced CKD, where inflammatory markers may be chronically elevated with limited biological variability, thereby reducing the ability to detect stronger correlations [42]. Furthermore, MDPs may exert context-dependent metabolic and cytoprotective effects that are not directly linked to measurable systemic cytokine release [43]. AGE–RAGE signaling may also contribute to tissue injury through intracellular oxidative pathways and mitochondrial reactive oxygen species generation in addition to systemic inflammatory mechanisms, which may not consistently translate into measurable changes in conventional inflammatory biomarkers [44]. Therefore, while the observed association between CRP and the AGEs/sRAGE ratio supports an interaction between inflammatory and AGE–RAGE pathways, the overall findings highlight the multifactorial nature of CKD pathophysiology and suggest that oxidative stress, mitochondrial dysfunction, and inflammation represent interconnected but only partially overlapping biological processes.

We also explored the potential relationships among AGEs, RAGE isoforms, MDPs and PEW. Neither MDPs nor AGEs were associated with PEW, and among RAGE isoforms, only esRAGE showed a univariate association that was not retained after multivariable adjustment. Although this finding may suggest a link between PEW and altered AGE–RAGE regulation, rather than to circulating AGEs or MDPs, it should be interpreted cautiously and confirmed in larger longitudinal studies. Overall, these findings likely reflect the multifactorial nature of PEW in CKD, which is driven by inflammation, metabolic acidosis, hormonal disturbances, reduced nutrient intake, dialysis-related factors, and muscle catabolism rather than a single biological pathway. Although AGE accumulation and mitochondrial dysfunction may contribute indirectly to metabolic derangements, circulating biomarkers may not reflect tissue-specific alterations, particularly within skeletal muscle, as principal site of protein loss in PEW. Indeed, skeletal muscle dysfunction and sarcopenia are highly prevalent in CKD and are strongly associated with mitochondrial impairment [45], and perhaps it is most likely that circulating MDP concentrations are not reflect local tissue expression or biological activity.

Several concerns and limitations should be acknowledged. First, the study cohort represents a highly selected subgroup of CKD-5 patients eligible for LD-KTx; therefore, the findings may not be generalizable to older patients, those on long-term dialysis, or patients with multiple comorbidities, and may not reflect the broader CKD population. Second, the older age of the control cohort represents a study limitation. Nevertheless, findings were largely unchanged in analyses restricted to younger controls. Third, the patients were heterogeneous with respect to dialysis status, dialysis modality, and dialysis duration. Fourth, the cross-sectional design precludes causal inference; therefore, the observed associations should not be interpreted as evidence of biologically linked or overlapping pathways. Fifth, the ELISA assay measured total circulating AGEs rather than individual AGE species, such as carboxymethyllysine (CML), carboxyethyllysine (CEL) and pentosidine, which may vary in their biological activity and affinity for RAGE; therefore, the present findings cannot distinguish the potentially distinct effects of specific AGE compounds. Sixth, circulating biomarkers may not accurately reflect tissue-specific mitochondrial or inflammatory activity. In addition, biomarkers were assessed at a single time point, which may not capture biological variability over time, and data on dietary AGE intake, which may have a potential influence on circulating AGE concentrations, were not available. Finally, residual confounding and assay-related variability inherent to immunoassay-based measurements cannot be excluded. Moreover, because multiple exploratory analyses were conducted, the probability of type I error is increased; therefore, these findings should be interpreted as hypothesis-generating rather than confirmatory. Despite these limitations, the study has several strengths, including the simultaneous evaluation of AGEs, multiple sRAGE isoforms, and several mitochondrial-derived peptides in a well-characterized CKD cohort. The findings provide novel clinical evidence supporting a biological interaction between glycoxidative stress and mitochondrial signaling pathways in CKD.

In conclusion, CKD-5 patients demonstrated increased levels of AGE-related biomarkers together with reduced MDPs, suggesting the presence of enhanced glycoxidative stress and impaired mitochondrial function. The associations between AGEs, RAGE isoforms, and MDPs suggest a pathophysiological link between glycation pathways and mitochondrial stress responses in CKD. These findings are consistent with the concept that the AGE–RAGE axis and mitochondrial dysfunction are biologically interconnected processes that may contribute to CKD-related metabolic disturbances [43]. While AGEs, RAGE isoforms, and MDPs were not consistently associated with PEW or most inflammatory markers, the AGEs/sRAGE ratio showed an independent positive association with CRP, supporting a link between AGE–RAGE pathway activity and systemic inflammation. Nevertheless, the overall pattern of weak and selective inflammatory associations suggests that these circulating biomarkers may not serve as direct indicators of nutritional status or generalized systemic inflammation in patients with kidney failure. Rather, they may reflect complementary aspects of CKD pathophysiology related to oxidative stress, mitochondrial dysfunction, and inflammatory signaling. Future longitudinal studies, including assessments following kidney transplantation, are needed to clarify the relationships between these biomarkers, renal function recovery, persistent metabolic abnormalities, and the effects of immunosuppressive therapy. In addition, mechanistic studies are warranted to determine whether targeting mitochondrial dysfunction and/or AGE-RAGE signaling could improve metabolic and inflammatory disturbances in CKD. Although the current role of these biomarkers is primarily investigational, future studies may determine whether they can serve as biomarkers of disease severity, risk stratification, prognosis, and treatment response in CKD. In addition, combined assessment of AGE-related biomarkers and MDPs may provide complementary information on glycoxidative stress and mitochondrial dysfunction that is not captured by conventional measures of kidney function or inflammation. Further longitudinal and mechanistic studies are required to establish their clinical relevance and predictive value.

## Figures and Tables

**Table 1:** Baseline characteristics of the CKD cohort (n = 160).

Variables	
Age, years	47 [32–58]
Males, n [%]	109 [68%]
Diabetes mellitus, n (%)	17 [11%]
Cardiovascular disease, n (%)	26 [16]
Protein-energy wasting, n (%)	40 [25%]
Body mass index, kg/m^2^	24.4 [22.3–26.5]
Estimated glomerular filtration rate, mL/min/1.73 m^2^	6.4 [5.2–8.4]
s-Albumin, g/dL	35 [32–38]
s-Creatinine, μmol/L	732 [589–870]
Hemoglobin, g/L	113 [105–122]
Parathyroid hormone, pg/mL	259 [160–400]
C-reactive protein, mg/dL	0.82 [0.34–2.20]
Interleukin-6, pg/mL	1.02 [0.49–2.01]
Tumor necrosis factor, pg/mL	10.6 [8.6–13.7]

Data presented as median [IQR, interquartile range].

**Table 2:** Spearman correlation matrix between AGEs, RAGE isoforms and MDPs.

	AGEs	sRAGE	esRAGE	cRAGE	HN	MOTS-c	HN-L1	AGEs /sRAGE
sRAGE	0.28 ^b^							
esRAGE	−0.15	−0.07						
cRAGE	0.25 ^a^	0.99 ^d^	−0.18					
HN	0.41 ^d^	0.30 ^b^	−0.17	0.35 ^b^				
MOTS-c	−0.22 ^b^	0.18	−0.05	0.16	−0.22 ^a^			
HN-L1	−0.12	−0.08	−0.03	−0.06	−0.33 ^c^	0.28 ^c^		
AGEs/sRAGE	−0.03	−0.94 ^d^	0.05	0.93 ^d^	−0.21 ^a^	−0.20 ^a^	0.12	
cRAGE/esRAGE	0.23 ^a^	0.90 ^d^	−0.42 ^d^	0.94 ^d^	0.39 ^c^	0.22 ^a^	−0.01	−0.87 ^d^

Data presents as Rho [ρ]. Significance levels: ^a^ <0.05, ^b^ <0.01, ^c^ <0.001 and ^d^ <0.0001. Abbreviations: AGEs, advanced glycation end products; sRAGE, soluble AGEs receptor; MDPs, mitochondrial-derived peptides; esRAGE, endogenous secretory RAGE; cRAGE, cleavage RAGE; HN, humanin; MOTS-c, mitochondrial open reading frame of the 12S rRNA-c; HN-L1, humanin-like 1.

**Table 3:** Multivariable linear regression model evaluating the determinants of esRAGE in CKD-5 patients.

Covariates	β	SE	t-Value	*p*-Value	95% CI	
Age	−0.001	0.006	−0.23	0.822	−0.014	0.011
Sex	0.052	0.180	0.29	0.770	−0.303	0.408
PEW	0.323	0.200	1.61	0.109	−0.073	0.720
CRP	0.003	0.009	0.27	0.784	−0.016	0.021
BMI	−0.038	0.026	−1.43	0.156	−0.090	0.015
eGFR	−0.038	0.034	−1.12	0.266	−0.106	0.030

Abbreviations: esRAGE, endogenous secretory RAGE; PEW, Protein-energy wasting; CRP, C-reactive protein; BMI, Body mass index; eGFR, estimated glomerular filtration rate.

## Data Availability

Data described in the manuscript, code book, and analytic code will be made available upon reasonable request pending application to the corresponding author.

## Supplementary Materials

The following supporting information can be downloaded at: https://www.mdpi.com/article/10.3390/antiox15080956/s1, Table S1: Comparison between patients included in the study and those not included from the cohort of CKD stage 5 patients undergoing living-donor renal transplantation.; Table S2a: Comparisons of clinical and biochemical characteristics and serum concentrations of AGEs, sRAGE isoforms and mitochondrial-derived peptides between control subjects and CKD stage 5 patients undergoing living-donor renal transplantation.; Table S2b: Comparison between younger control subjects (below the median age) and CKD-5 patients.; Table S3: Comparisons of serum concentrations of AGEs, sRAGE isoforms and MDPs between dialyzed and non-dialyzed living donors-kidney transplant CKD-5 patients.; Table S4a: Age correlations with AGEs, sRAGE isoforms, and mitochondrial-derived peptides (HN, MOTS-c and HN-L1) in living donors-kidney transplant CKD-5 patients and control subjects.; Table S4b: Sex-related differences in serum concentrations of AGEs, sRAGE isoforms, and mitochondrial-derived peptides (MOTS-c, HN and HN-L1) in living donors-kidney transplant CKD-5 patients and control subjects.; Table S5a: Multivariate linear regression analysis of factors associated with serum AGEs concentration in living-donor kidney transplant CKD-5 patients.; Table S5b: Multivariate linear regression analysis of factors associated with serum sRAGE concentration in living-donor kidney transplant CKD-5 patients.; Table S5c: Multivariate linear regression analysis of factors associated with AGEs/sRAGE ratio in living-donor kidney transplant CKD-5 patients.; Table S6a: Multivariate linear regression analysis of factors associated with serum AGEs concentration in living-donor kidney transplant CKD-5 patients.; Table S6b: Multivariate linear regression analysis of factors associated with serum sRAGE concentration in living-donor kidney transplant CKD-5 patients.; Table S6c: Multivariate linear regression analysis of factors associated with AGEs/sRAGE ratio in living-donor kidney transplant CKD-5 patients.
