# Supplementary material for: AGEs–RAGE Axis and Mitochondrial-Derived Peptides in Advanced Chronic Kidney Disease: Interconnected Pathways Beyond Inflammation and Nutrition
Source: Antioxidants (Basel). 2026 Jul 30;15(8):956. doi: 10.3390/antiox15080956 (PMC13510039; doi:10.3390/antiox15080956)
Supplement: Supplementary file 1 [file antioxidants-15-00956-s001.zip › antioxidants-4414087-supplementary.pdf]

Supplementary materials:

Table S1. Comparison between patients included in the study and those not included from the cohort of CKD stage 5 patients undergoing living-donor renal transplantation.

Variables	Included patients	Excluded patients	P-value
Number	160	45	
Age, years	47 (32-58)	47 (36-54)	0.76
Males, n (%)	109 (68%)	32 (71%)	0.59
Diabetes mellitus, n (%)	17 (11%)	4 (9%)	0.64
Cardiovascular disease, n (%)	26 (16)	3 (7%)	0.03
Protein energy wasting, n (%)	40 (25%)	13 (28%)	0.67
Body mass index, kg/m^2	24.4 (22.3-26.5)	24.0 (22.7-27.2)	0.75
eGFR, ml/min/1.73m^2	6.4 (5.2-8.4)	5.8 (4.2-7.8)	0.43
s-Albumin, g/dL	35 (32-38)	35 (31-38)	0.46
s-Creatinine, µmol/L	732 (589-870)	722 (592-903)	0.83
Hemoglobin, g/L	113 (105-122)	143 (132-146)	<0.0001
PTH, pg/mL	259 (160-400)	235 (180-290)	0.76
C-reactive protein, mg/dL	0.82 (0.34-2.20)	1.00 (0.36-2.30)	0.61

Data presented as median (IQR) or n= (%).

Table S2a. Comparisons of clinical and biochemical characteristics and serum concentrations of AGEs, sRAGE isoforms and mitochondrial-derived peptides between control subjects and CKD stage 5 patients undergoing living-donor renal transplantation.

Variables	Patients (n=160)	Control (n=80)	p-value
Age, years	47 (32-58)	63 (55-70)	<0.0001
Males, n (%)	109 (68%)	57 (71%)	0.62
Diabetes mellitus, n (%)	17 (11%)	4 (5%)	0.25
Cardiovascular disease, n (%)	26 (16%)	7 (9 %)	0.27
Protein energy wasting, n (%)	40 (25%)	2 (2.5%)	<0.0001
Body mass index, kg/m^2	24.4 (22.3-26.5)	25.4 (23.3-28.3)	<0.05
eGFR, ml/min/1.73m^2	6.4 (5.2-8.4)	81.3 (75.0-97.3)	<0.0001
s-Albumin, g/dL	35 (32-38)	39 (37-41)	<0.0001
s-Creatinine, µmol/L	732 (589-870)	79 (69-91)	<0.0001
Hemoglobin, g/L	113 (105-122)	144 (136-149)	<0.0001
PTH, pg/mL	259 (160-400)	37.0 (29.8-49.0)	<0.0001
C-reactive protein, mg/dL	0.82 (0.34-2.20)	1.3 (0.7-2.8)	0.003
Interleukin-6, pg/mL	1.02 (0.49-2.01)	1.3 (0.99-2.7)	0.03
TNF, pg/mL	10.6 (8.6-13.7)	3.9 (3.1-5.4)	<0.001
AGEs, ng/ml	607 (501-759)	537 (451-615)	0.015
sRAGE, pg/ml	2030 (537-3662)	1624 (287-5192)	0.18
AGEs/sRAGE, ratio	0.285 (0.130-0.801)	0.424 (0.092-2.719)	0.013
esRAGE, pg/ml	227 (168-401)	167 (126-235)	<0.001
cRAGE, pg/ml	1749 (301-3403)	1462 (45-5163)	0.41
Humanin, pg/ml	2054 (1709-2658)	3104 (2440-4025)	<0.001
MOTS-c, pg/ml	113 (75-160)	141 (110-183)	<0.001
Humanin -like1, ng/ml	4.16 (3.07-6.13)	4.75 (3.60-6.50)	<0.001

Data presented as median (IQR) or n= (%). Abbreviations: eGFR, estimated glomerular filtration rate; AGEs, advanced glycation end products; sRAGE, soluble AGEs receptor; esRAGE¸ endogenous secretory RAGE; cRAGE, cleavage RAGE; MOTS-c, mitochondrial open reading frame of the 12S rRNA-c.

Table S2b. Comparison between younger control subjects (below the median age) and CKD-5 patients.

Variables	Patients (n=160)	Younger control (n=40)	p-value
Age, years	47 (32-58)	55 (48-60)	0.001
Males, n (%)	109 (68%)	26 (65%)	0.72
AGEs, ng/ml	607 (501-759)	538 (463-616)	0.04
sRAGE, pg/ml	2030 (537-3662)	2135 (383-4960)	0.75
AGEs/sRAGE ratio	0.285 (0.130-0.801)	0.264 (0.182-1.280)	0.48
esRAGE, pg/ml	227 (168-401)	173 (125-230)	<0.001
cRAGE, pg/ml	1749 (301-3403)	1890 (240-4915)	0.93
Humanin, pg/ml	2054 (1709-2658)	3522 (2452-4268)	<0.001
MOTS-c, pg/ml	113 (75-160)	126 (92-173)	0.14
Humanin-like 1, ng/ml	4.90 (3.88-6.38)	4.16 (3.07-6.13)	0.03

Data presented as median (IQR) or n=(%). Abbreviations: AGEs, advanced glycation end products; sRAGE, soluble AGEs receptor; esRAGE¸ endogenous secretory RAGE; cRAGE, cleavage RAGE; MOTS-c, mitochondrial open reading frame of the 12S rRNA-c.

Table S3. Comparisons of serum concentrations of AGEs, sRAGE isoforms and MDPs between dialyzed and non-dialyzed living donors-kidney transplant CKD-5 patients

Variables	Non-dialyzed	Dialyzed	P-value
Male, n (%)	35 (65%)	74 (69%)	0.52
Age, year	46 (35-57)	49 (32-58)	0.70
AGEs, ng/ml	609 (508-716)	602 (467-812)	0.99
sRAGE, pg/ml	2600 (744-10943)	1967 (506-3508)	0.22
AGEs/RAGE, ratio	0.08 (0.04-0.75)	0.31 (0.15-1.02)	0.25
esRAGE, pg/ml	228 (158-298)	242 (177-450)	0.16
cRAGE, pg/ml	2249 (537-10623)	1718 (220-2904)	0.11
HN, pg/ml	1923 (1671-2718)	2084 (1721-2619)	0.69
MOTS-c, pg/ml	109 (74-160)	114 (75-161)	0.92
HN-L1, ng/ml	4.22 (3.15-6.40)	4.04 (3.00-6.10)	0.62

Data presented as n (%) or median (IQR). Abbreviations: AGEs, advanced glycation end products; sRAGE, soluble AGEs receptor; MDPs, mitochondrial-derived peptides; esRAGE¸ endogenous secretory RAGE; cRAGE, cleavage RAGE; HN, humanin; MOTS-c, mitochondrial open reading frame of the 12S rRNA-c: HN-L1, humanin-like 1.

Table S4a: Age correlations with AGEs, sRAGE isoforms, and mitochondrial-derived peptides (HN, MOTS-c and HN-L1) in living donors-kidney transplant CKD-5 patients and control subjects.

**Variables**	**CKD patients**		**Control subjects**	
	ρ	p-value	ρ	p-value
AGEs	0.06	0.44	-0.08	0.51
sRAGE	0.04	0.68	-0.15	0.28
AGEs/sRAGE ratio	-0.04	0.69	0.39	0.008
esRAGE	-0.13	0.12	-0.01	0.93
cRAGE	0.01	0.99	-0.15	0.27
HN	-0.09	0.13	-0.24	0.04
MOTS-c	0.05	0.51	0.25	0.03
HN-L1	-0.13	0.09	-0.06	0.60

Abbreviations: ρ, rho: AGEs, advanced glycation end products; sRAGE, soluble AGEs receptor; esRAGE¸ endogenous secretory RAGE; cRAGE, cleavage RAGE; HN, humanin; MOTS-c, mitochondrial open reading frame of the 12S rRNA-c; HN-L1, humanin-like 1

Table S4b: Sex-related differences in serum concentrations of AGEs, sRAGE isoforms, and mitochondrial-derived peptides (MOTS-c, HN and HN-L1) in living donors-kidney transplant CKD-5 patients and control subjects.

**Patients**	**AGEs**	**sRAGE**	**AGEs/sRAGE**	**esRAGE**	**cRAGE**	**MOTs**	**Humanin**	**Humanin-L1**
**Males**	596 (511-782)	2029 (483-3585)	0.298 (0.149-0.988)	226 (176-419)	1741 (319-3382)	112 (74-153)	2187 (1712-2780)	3.96 (3.11-5.96)
**Females**	609 (472-742)	2052 (871-15462)	0.276 (0.035-0.665)	236 (158-337)	1756 (251-11312)	114 (77-441)	1970 (1705-2267)	4.67 (2.93-7.44)
p-value	0.73	0.37	0.25	0.66	0.85	0.27	0.19	0.38
**Controls**	**AGEs**	**sRAGE**	**AGEs/sRAGE**	**esRAGE**	**cRAGE**	**MOTs**	**Humanin**	**Humanin-L1**
**Males**	520 (442-598)	727 (164-3231)	0.999 (0.223-3.481)	185 (142-271)	423 (59-3572)	140 (109-174)	2830 (2367-3847)	4.93 (3.74-6.57)
**Females**	578 (505-711)	4193 (1639-5685)	0.264 (0.151-0.402)	137 (96-186)	4025 (1495-5451)	141 (110-228)	3694 (3026-4579)	4.07 (3.40-6.16)
p-value	0.02	0.03	0.03	0.006	0.02	0.45	0.01	0.35

Abbreviations: AGEs, advanced glycation end products; sRAGE, soluble AGEs receptor; esRAGE¸ endogenous secretory RAGE; cRAGE, cleavage RAGE; HN, humanin; MOTS-c, mitochondrial open reading frame of the 12S rRNA-c; HN-L1, humanin-like 1

Table S5a. Multivariate linear regression analysis of factors associated with serum AGEs concentration in living-donor kidney transplant CKD-5 patients.

Covariates	β	SE	t-value	p-value	95% CI	
**Humanin**	0.350	0.087	4.00	**<0.0001**	0.176	0.523
Age	-0.004	0.007	-0.66	0.513	-0.018	0.009
Sex	-0.049	0.184	-0.27	0.791	-0.413	0.316
CRP	0.005	0.008	0.55	0.583	-0.012	0.022
PEW	0.303	0.223	1.36	0.178	-0.140	0.746
BMI	0.005	0.031	0.16	0.872	-0.057	0.067
eGFR	-0.016	0.032	-0.49	0.628	-0.081	0.049

Abbreviations: AGEs, advanced glycation end products; CRP, C-reactive protein; PEW, Protein-energy wasting; BMI, Body mass index; eGFR, estimated glomerular filtration rate.

Table S5b. Multivariate linear regression analysis of factors associated with serum sRAGE concentration in living-donor kidney transplant CKD-5 patients.

Covariates	β	SE	t-value	p-value	95% CI	
**Humanin**	0.299	0.097	3.09	**0.003**	0.106	0.492
Age	0.004	0.008	0.50	0.618	-0.012	0.021
Sex	-0.262	0.212	-1.24	0.220	-0.685	0.161
CRP	-0.135	0.011	-1.20	0.235	-0.036	0.009
PEW	-0.131	0.278	-0.47	0.638	-0.686	0.424
BMI	0.013	0.038	0.34	0.734	-0.063	0.090
eGFR	0.034	0.035	0.97	0.335	-0.036	0.104

Abbreviations: sRAGE, soluble AGEs receptor; CRP, C-reactive protein; PEW, Protein-energy wasting; BMI, Body mass index; eGFR, estimated glomerular filtration rate.

Table S5c. Multivariate linear regression analysis of factors associated with AGEs/sRAGE ratio in living-donor kidney transplant CKD-5 patients.

Covariates	β	SE	t-value	p-value	95% CI	
**Humanin**	-0.251	0.101	-2.46	**0.016**	-0.454	-0.048
Age	-0.004	0.008	-0.43	0.671	-0.021	0.014
Sex	0.274	0.224	1.23	0.224	-0.172	0.721
CRP	0.015	0.012	1.25	0.217	-0.009	0.038
PEW	0.224	0.293	0.83	0.409	-0.341	0.829
BMI	-0.012	0.040	-0.29	0.770	-0.092	0.069
eGFR	-0.040	0.037	-1.08	0.282	-0.115	0.034

Abbreviations: AGEs/sRAGE, advanced glycation end products /soluble AGEs receptor; CRP, C-reactive protein; PEW, Protein-energy wasting; BMI, Body mass index; eGFR, estimated glomerular filtration rate.

Table S6a. Multivariate linear regression analysis of factors associated with serum AGEs concentration in living-donor kidney transplant CKD-5 patients.

Covariates	β	SE	t-value	p-value	95% CI	
**MOTS-c**	-0.112	0.087	-1.29	0.201	-0.284	0,060
Age	0.002	0.006	0.30	0.768	-0.011	0.015
Sex	0.170	0.190	0.89	0.375	-0.207	0.546
CRP	-0.001	0.010	-0.05	0.960	-0.020	0.019
PEW	0.367	0.226	1.62	0.107	-0.080	0.814
BMI	-0.023	0.029	-0,79	0.429	-0.080	0.034
eGFR	-0.039	0.035	-1.11	0.268	-0.109	0.031

Abbreviations: AGEs, advanced glycation end products; CRP, C-reactive protein; PEW, Protein-energy wasting; BMI, Body mass index; eGFR, estimated glomerular filtration rate.

Table S6b. Multivariate linear regression analysis of factors associated with serum sRAGE concentration in living-donor kidney transplant CKD-5 patients.

Covariates	β	SE	t-value	p-value	95% CI	
**MOTS-c**	0.182	0.092	1.99	**0.049**	0.001	0.364
Age	0.010	0.007	1.30	0.197	-0.005	0.025
Sex	-0.268	0.221	-1.22	0.227	-0.706	0.170
CRP	-0.014	0.012	-1.14	0.255	-0.039	0.010
PEW	-0.255	0.249	-1.02	0.309	-0.750	0.240
BMI	-0.045	0.035	-1.26	0.210	-0.115	0.026
eGFR	0.029	0.037	0.77	0.445	-0.045	0.102

Abbreviations: sRAGE, soluble AGEs receptor; MOTS-c, mitochondrial open reading frame of the 12S rRNA-c; CRP, C-reactive protein; PEW, Protein-energy wasting; BMI, Body mass index; eGFR, estimated glomerular filtration rate.

Table S6c. Multivariate linear regression analysis of factors associated with AGEs/sRAGE ratio in living-donor kidney transplant CKD-5 patients.

Covariates	β	SE	t-value	p-value	95% CI	
**MOTS-c**	-0.219	0.093	-2.35	**0.021**	-0.405	-0.034
Age	-0.173	0.112	-1.55	0.125	-0.397	0.049
Sex	0.301	0.224	1.35	0.182	-0.144	0.748
CRP	0.244	0.121	2.02	**0.047**	0.004	0.484
PEW	0.137	0.270	0.51	0.613	-0.400	0.674
BMI	0.064	0.132	0.49	0.629	-0.198	0.326
eGFR	-0.076	0.103	-0.73	0.465	-0.283	0.130

Abbreviations: AGEs/sRAGE, advanced glycation end products /soluble AGEs receptor; MOTS-c, mitochondrial open reading frame of the 12S rRNA-c; CRP, C-reactive protein; PEW, Protein-energy wasting; BMI, Body mass index; eGFR, estimated glomerular filtration rate.
